# Supplementary figures and images for: Veterinary trypanocidal benzoxaboroles are peptidase-activated prodrugs
Source: PLoS Pathog. 2020 Nov 3;16(11):e1008932. doi: 10.1371/journal.ppat.1008932 (PMC7710103; doi:10.1371/journal.ppat.1008932)

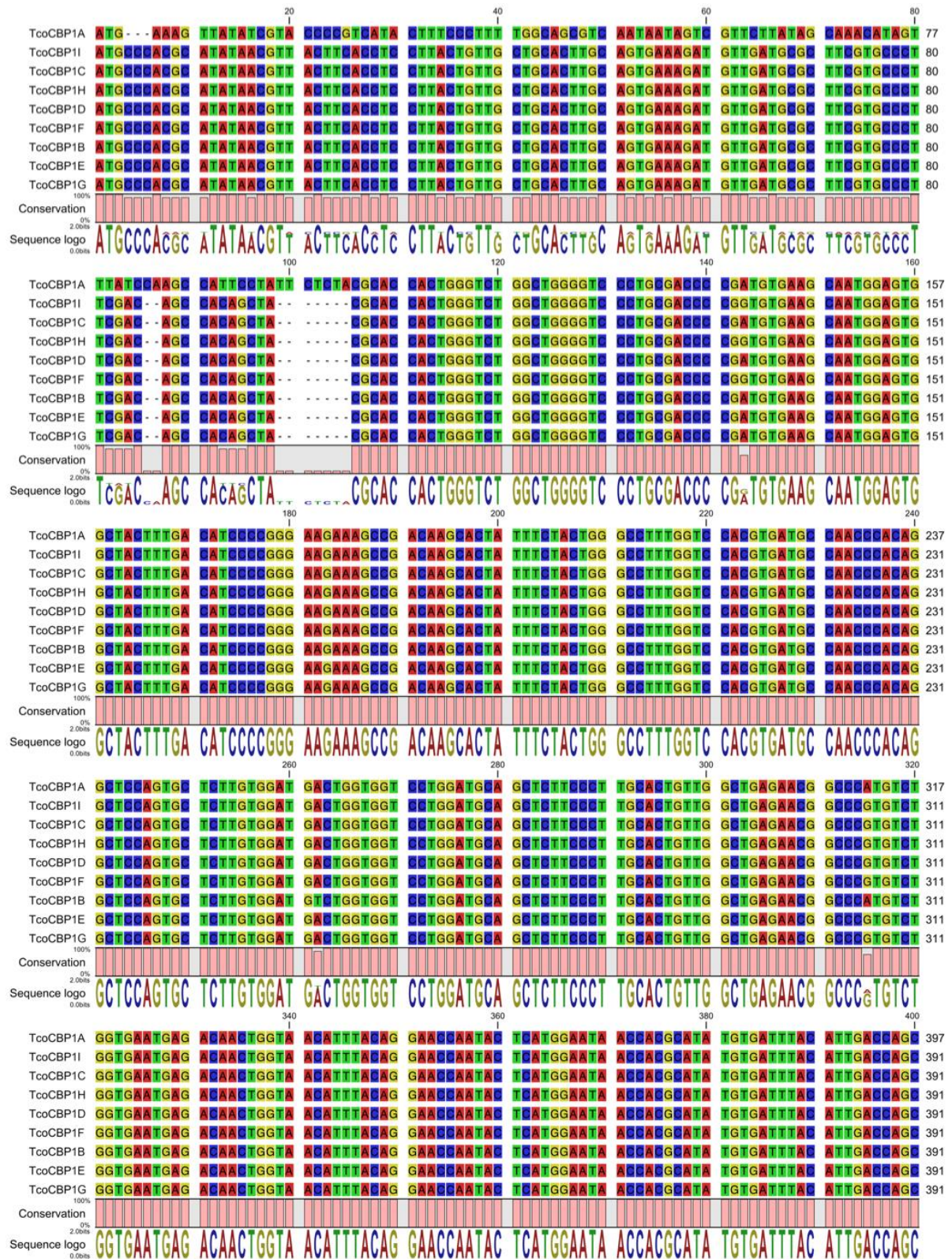

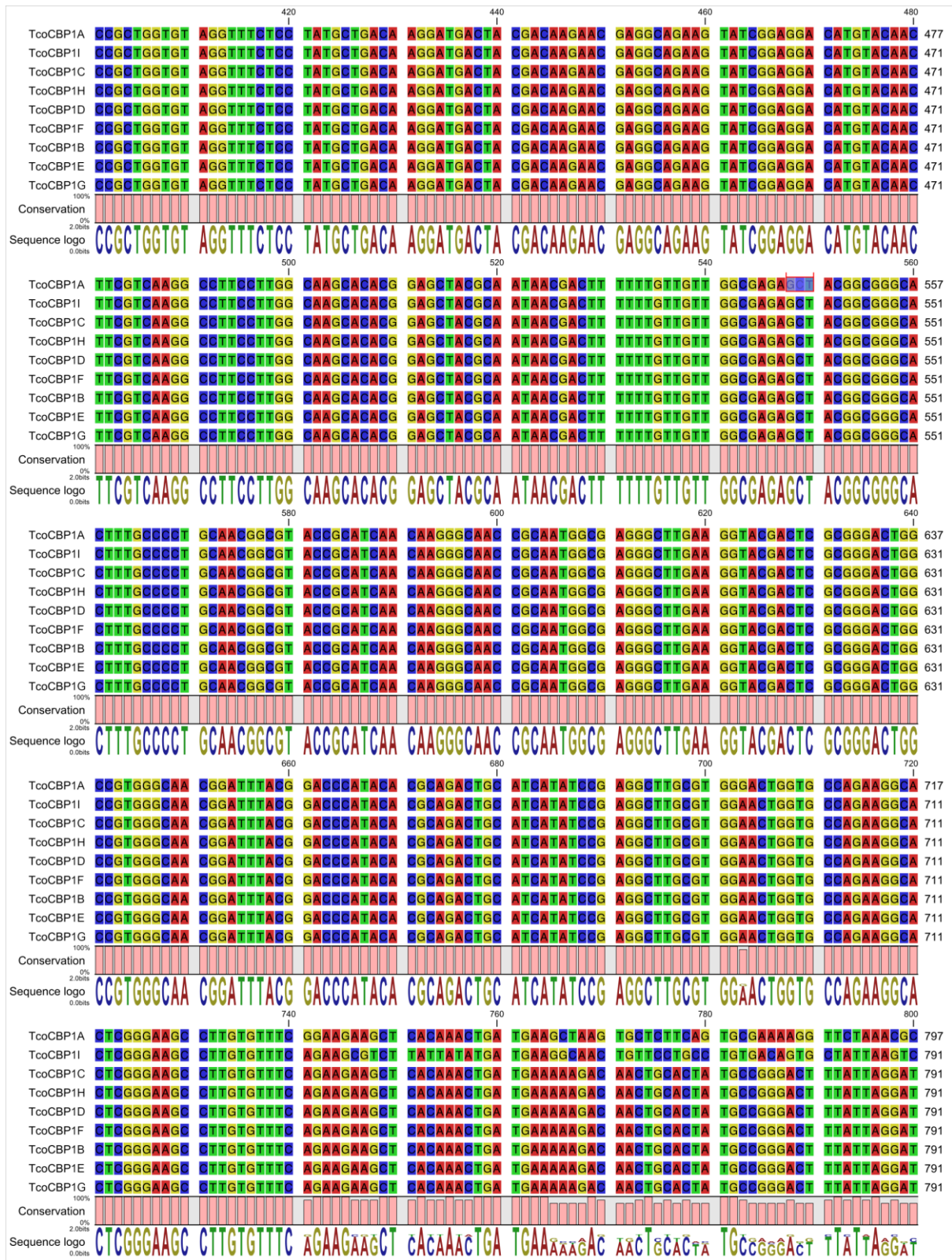

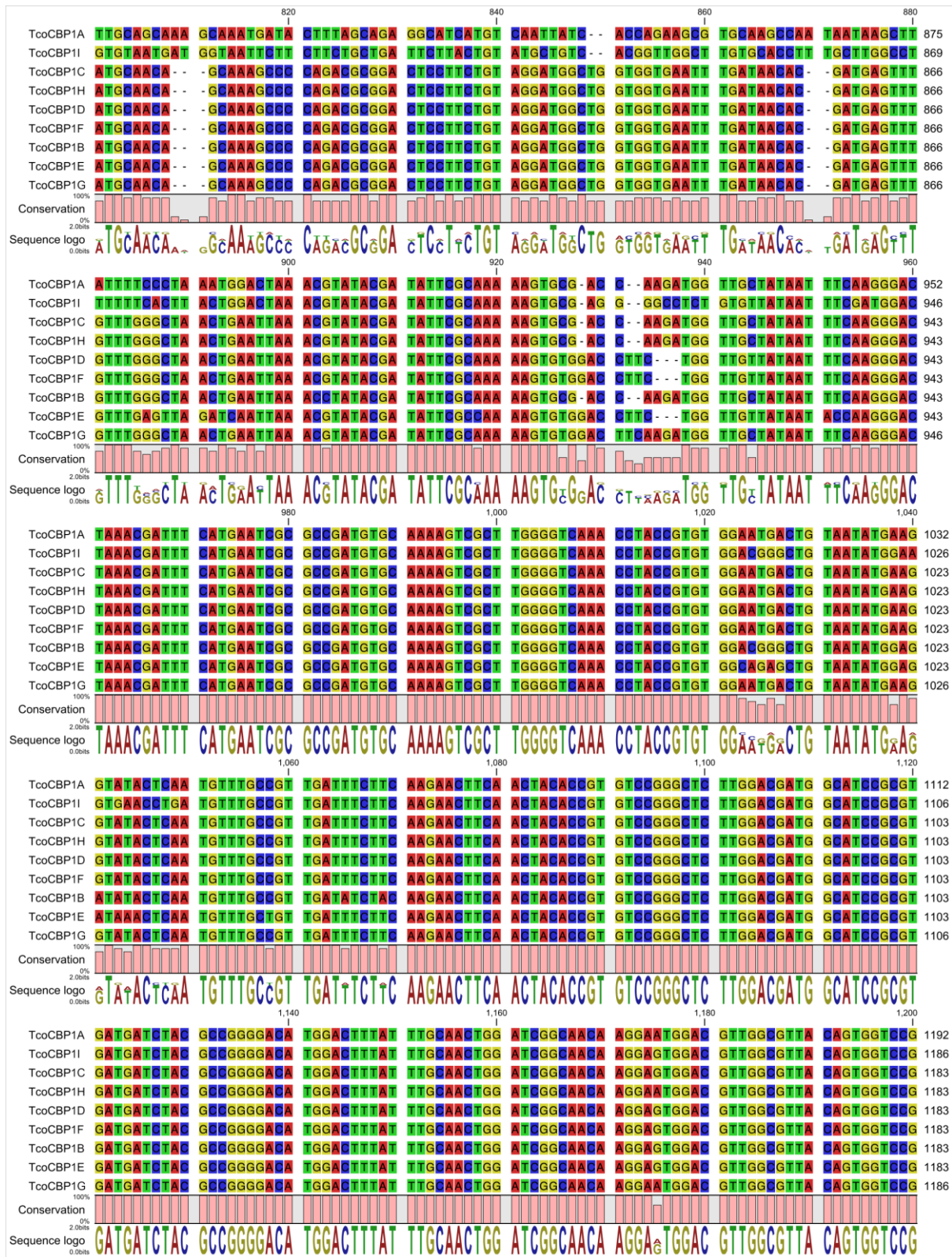

Supplement: S2 Fig — The alignment was made with CLC genomics workbench using the TcIL3000 reference genome available from TriTrypDB (https://tritrypdb.org/tritrypdb/). (PDF) [file ppat.1008932.s002.pdf]

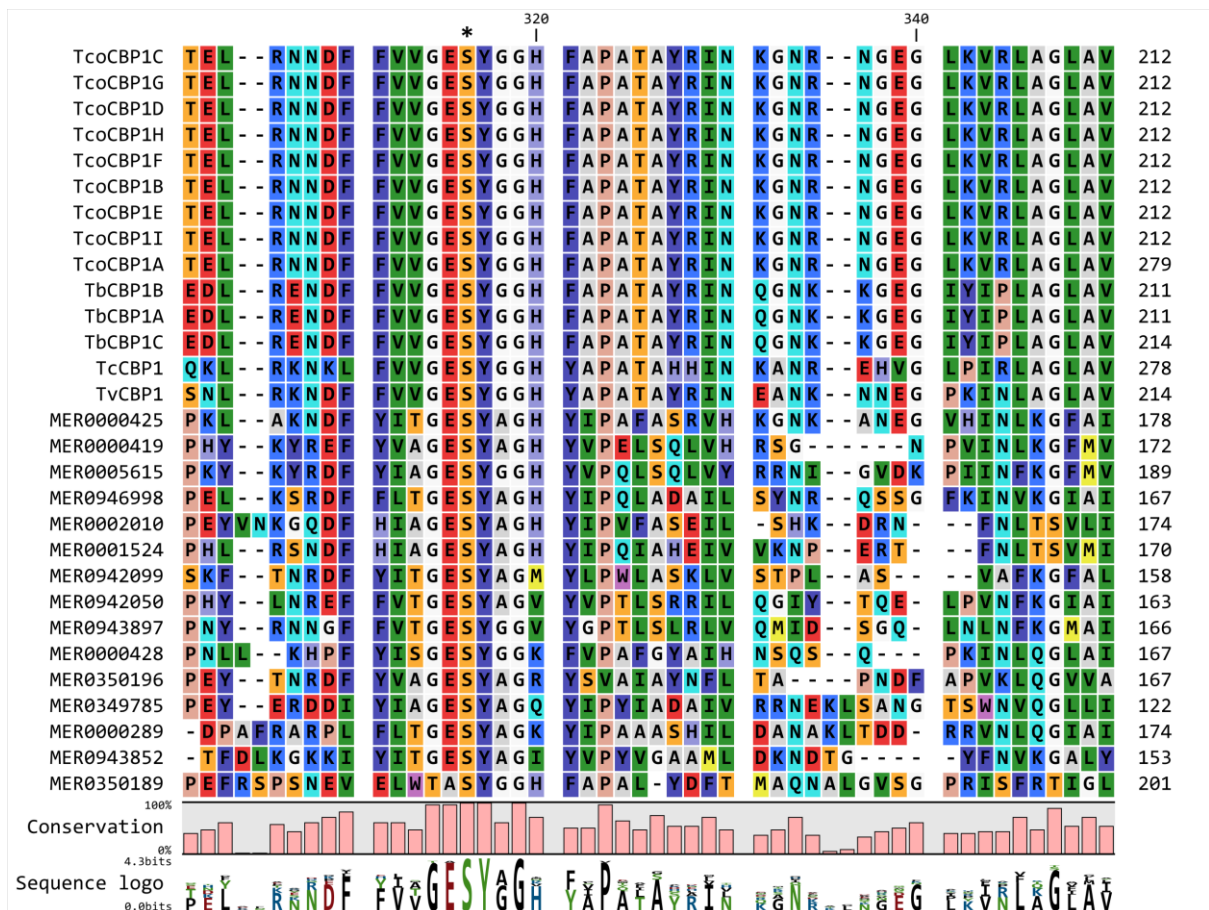

Supplement: S4 Fig — Extract of alignment of the annotated serine carboxypeptidases from T. congolense (TcoCPB1A-I), T. b. brucei (TbCBP1A-C, Tbb427.10.1030–50), T. vivax (TvCBP1, TvY486_1000990) and T. cruzi (TcCBP1, TcCLB.508671.20). The sequences were blasted against the entire collection of S10 carboxypeptidases stored at MEROPS Peptidase Database [42]. Family S10 has residues of the catalytic triad in the order Ser, Asp and His [29, 30] and carboxypeptidase Y (MER0002010) from Saccharomyces cerevisiae is the most representative gene of the family. Indicated with asterisks is the polar catalytic serine (S179) of the triad. This Ser was targeted in Tb927.10.1040 for site directed mutagenesis, substituting with a hydrophobic alanine (S179A). The alignment was made with CLC genomics workbench. (PDF) [file ppat.1008932.s004.pdf]

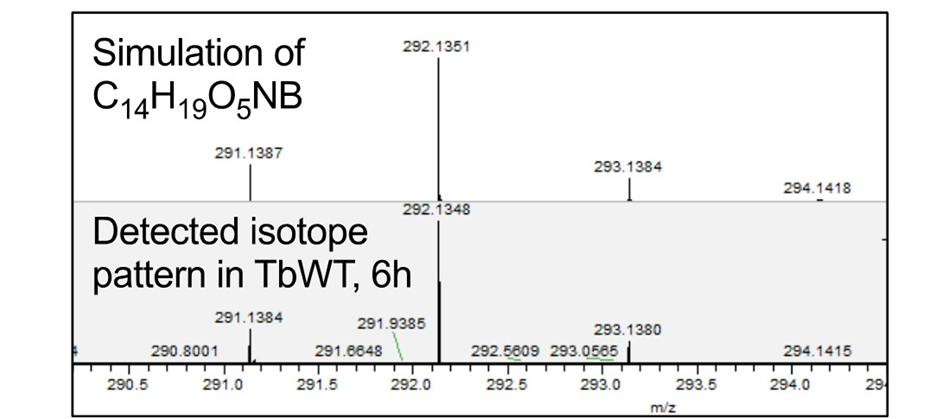

Supplement: S5 Fig — The fragment had an isotopic distribution that matched the simulated isotopic distribution of C14H19O5NB (example obtained for a TbWT replicate treated for 6 h with AN11736). (TIFF) [file ppat.1008932.s005.tiff]

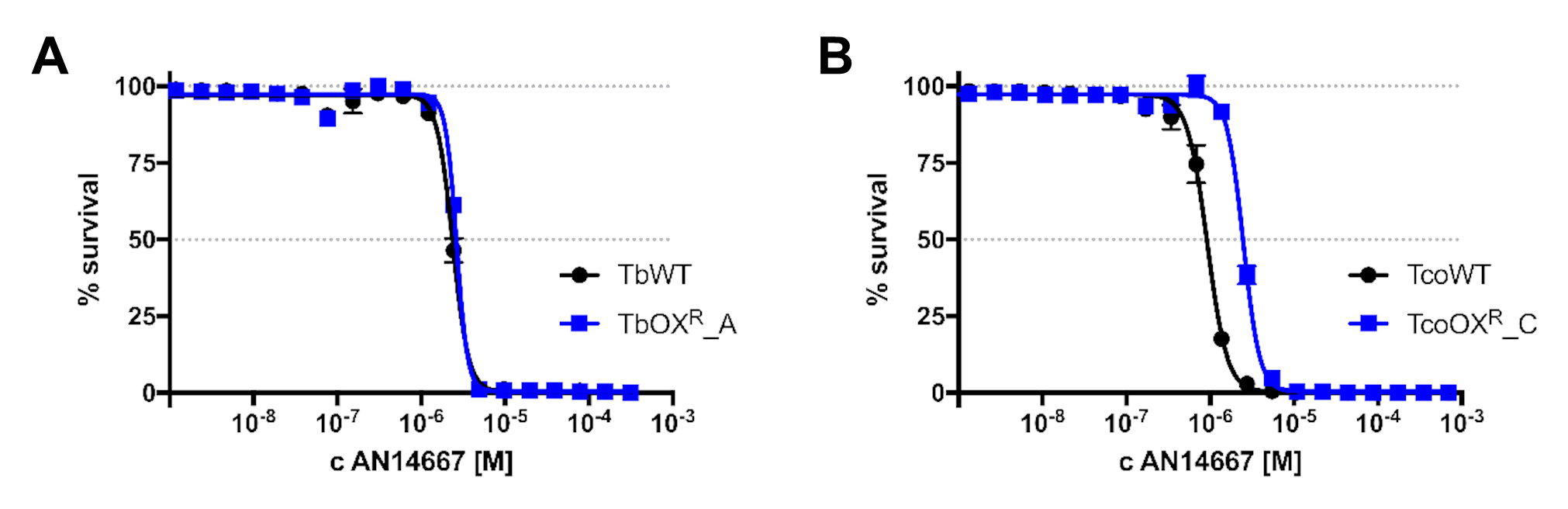

Supplement: S6 Fig — (A) No difference in susceptibility to AN14667 was found for TbWT and the resistant line TbOXR_A (EC50 9.5 μM and 10.55 μM respectively). (B) The AN11736 resistant T. congolense line TcoOXR_C was more than 2.5-fold more resistant to the metabolite than TcoWT (EC50 2.49 μM and 0.92 μM respectively); Data represent means ± SD of n = 3 independent biological replicates. (TIFF) [file ppat.1008932.s006.tiff]
