# Supplementary material for: Veterinary trypanocidal benzoxaboroles are peptidase-activated prodrugs
Source: PLoS Pathog. 2020 Nov 3;16(11):e1008932. doi: 10.1371/journal.ppat.1008932 (PMC7710103; doi:10.1371/journal.ppat.1008932)
Supplement: S3 Fig — (A) Nucleotide sequence. (B) Translated amino acid sequence. In bold, identical to only Tb427.10.1050; bold and italicised, identical to only Tb427.10.1030; normal, no difference to Tb427.10.1040; italicised and underlined are differences that are not present in 1050 or 1030. Blue and underlined, identical to Tb427.10.1050 and Tb427.10.1040. Red, underlined and bold, unique to the chimera. Underlined sequence, Cas9 targeting sequence. (PDF) [file ppat.1008932.s003.pdf]

A

ATGCGGCTCATTTCGTATCCGGTAATGCTATCGTTACTGGCTGCTTGCATCC
TGGTCGTAGTTTTAGCAAATACTGTCTATCGTAGTTTATCTCTAGCGCTTCGC
ACGACGGGCTCTGGTTGGGAGCCTTGTGACCCCGGCGTTAATCAATGGAGCGGA
TATTTTGATATTCCTGGAGAGCAGAGTGACAAACATTACTTCTACTGGGCATTTG
GACCACGTGATGGCAATCCCAATGCCCCAGTGCTCCTGTGGATGACAGGTGGTCC
TGGATGTAGTTCCATG***C***TTGCGCTACTTGCAGAGAATGGACCTTGCCT*T*ATGAAC
GAAACGACTGGTGACATATACAACAATACGTACTCGTGGAACAACCACGCGTAT
GTAATATATATCGATCAACCTGCCGGTGTGGGCTTTTCGTATGCGGATAAAGCGG
ATTATGATAAGAATGAAGCGGAGGTATCGGAGGACATGTACAACTTTCTACAGG
CCTTCTTTGGTGAGCACGAGGACCTGCGTGAAAACGACTTCTTTGTTGTTGGGGA
AAGCTACGGTGGCCACTTTGCTCCGGCTACAGCTTACCGCATTAACCAAGGCAAT
AAAAAGGGTGAAGGCATATACATTCCTCTTGCAGGATTGGCTGTAGGAAACGGT
TTAACAGATCCGTATACACAATATGCGTCATATCCCAGACTTGCATGGGACTGGT
GCAAGGAAGTTCT***TGGAAGTCCGTGTGTATCTTCCTTTGTTCATGTGATGATGTCG***
GCGATGGTGCCCGCTTGTCAAAGTACTATTTCTGCTTGTGACGCTGATAATTCATC
ATCCGCGGACTCGTCGTGCAAGCTTTCCCGTGTGACTTGTGGTCCGATGGTAGCTC
TGTTTTCAGCTACAGGCCTGAACGTTTATGATATTCGTAAGCCATGTGATGGGCCA
TTATGTTACAACACAACGGGAGTTGACAACTTTATGAACCGTGAGGATGTTCAGAG
GTCTCTGGGTGTAGATCCAATGACTTGGCAAGCTTGTAACATGGAGGTGAACCTGA
TGTTTGCCGTTGACTGGTTTAAGAACTTTAACTACACAATTAGTGGACTCCTCGA
AGATGGAGTTCGAGTAATGATTTATGCCGGTGATATGGACTTTATCTGCAACTGG
ATTGGAAATAAGGAGTGGACACTTGCACTTCAGTGGTCTGGAAGTGAAGAGTTT
GTGAAGGCCCCTGATACCCCATTCTCATCTATTGATGGTAGTGCTGCGGGTCTTG
TACGTAGTGTATCATCAAACACATCGTCAATGCACTTCAGTTTTGTGCAGGTGTA
CCGTGCGGGTCACATGGTGCCGATGGATCAACCTGCTGCGGCATCCACTATAATT
GA**A**AAATTCATGAGGAACGAACCACTCTCGTAA

B

MRLISYPVMLSLLAACILVVVLANTVYRSLSLALRTTGSGWEPCDPGVNQWSGYF

DIPGE

QSDKHYFYWAFGPRDGNPNAPVLLWMTGGPGCSSM*L*ALLAENGPCLMNETTGDIY

NNTYS

WNNHAYVIYIDQPAGVGFSYADKADYDKNEAEVSEDMYNFLQAFFGEHEDLREND

FFVVG

ESYGGHFAPATAYRINQGNKKGEGIYIPLAGLAVGNGLTDPYTQYASYPRLAWDWC

KEVL

G***SPCVSSFVHVMMSAMVPACQSTISACDADNSSSADSSCKLSRVTCGPMVALFSATG***

LNV

YDIRKPCDGPLCYNTTGVDNFMNREDVQRSLGVDPMTWQACNMEVNLMFAVDWF

KNFNYT

ISGLLEDGVRVMIYAGDMDFICNWIGNKEWTLALQWSGSEEFVKAPDTPFSSIDGSA

AGL

VRSVSSNTSSMHFSFVQVYRAGHMVPMDQPAAASTIIEKFMRNEPLS
