# Supplementary material for: Veterinary trypanocidal benzoxaboroles are peptidase-activated prodrugs
Source: PLoS Pathog. 2020 Nov 3;16(11):e1008932. doi: 10.1371/journal.ppat.1008932 (PMC7710103; doi:10.1371/journal.ppat.1008932)
Supplement: S1 Table — (PDF) [file ppat.1008932.s007.pdf]

Compound	TcoWT	$TcoOX^R$_B		$TcoOX^R$_C	
	(EC_{50} nM ± SD) (*n*)	(EC_{50} nM ± SD) (*n*)	RF vs WT	(EC_{50} nM ± SD) (*n*)	RF vs WT
AN11736	0.46 ± 0.28 (*10*)	17.84 ± 5.99 (*7*)	39	37.24 ± 17.67 (*7*)	81
Acoziborole	511.70 ± 35.37 (*3*)	506.93 ± 25.75 (*3*)	1.0	ND	
Diminazene	220.03 ± 33.66 (*6*)	229.0 ± 21.74 (*3*)	1.0	195.20 ± 15.05 (*3*)	0.9
Pentamidine	701.40 ± 44.59 (*3*)	1059.83 ± 195.15 (*3*)	1.5	775.10 ± 115.52 (*3*)	1.1
Isometamidium	0.68 ± 0.26 (*4*)	1.15 ± 0.61 (*4*)	1.7	0.78 ± 0.08 (*4*)	1.1
Ethidium Bromide (Homidium)	9.16 ± 2.0 (*4*)	12.56 ± 5.58 (*4*)	1.4	12.57 ± 1.22 (*4*)	1.4
Suramin	22647.5 ± 1822.5 (*4*)	28827.5 ± 14932.7 (*4*)	1.3	23925.0 ± 1746.1 (*4*)	1.1
Eflornithine	27292.5 ± 1170.2 (*4*)	16974.0 ± 8217.2 (*4*)	0.6	27355.0 ± 1352.2 (*4*)	1.0

RF, resistance factor (ratio of the EC_{50} measured for the AN11736 resistant clones to the EC_{50} measured for the parental WT line, TcoWT). ND, not determined; *n*, number of independent biological replicates. Note that the EC_{50} value for AN11736 in this set of experiments slightly differs from the one presented in Fig. 1.
