# Supplementary material for: Veterinary trypanocidal benzoxaboroles are peptidase-activated prodrugs
Source: PLoS Pathog. 2020 Nov 3;16(11):e1008932. doi: 10.1371/journal.ppat.1008932 (PMC7710103; doi:10.1371/journal.ppat.1008932)
Supplement: S2 Table — (PDF) [file ppat.1008932.s008.pdf]

Compound	Structure	TcoWT	TcoOX^{R}_B[#]		TcoOX^{R}_C	
		EC_{50} nM	EC_{50} nM	RF vs WT	EC_{50} nM	RF vs WT
(a) AN14503	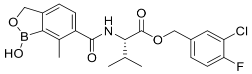	0.85 ± 0.77	25.55 ± 11.36	30	73.16 ± 28.57	87
(b) AN14707	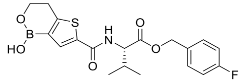	7.24 ± 3.84	387.4 ± 110.5	54	561.1 ± 91.9	78
(c) AN14353	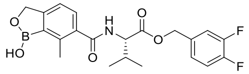	0.15 ± 0.12	15.24 ± 4.81	104	48.21 ± 25.69	328
(d) AN14725	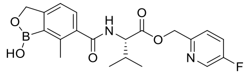	0.27 ± 0.13	8.43 ± 6.6	31	11.92 ± 0.95	43
(e) AN14728	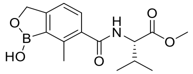	8.71 ± 0.73	706.1 ± 249.4	81	782. 5 ± 80.65	90
(f) AN15174	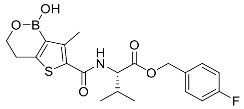	253 ±88.1	5615 ± 817	22	18127 ± 1956	72
(g) AN14337	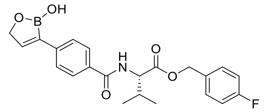	158.3 ± 70.2	405.1 ± 160.4	2.6	334.8 ± 27.1	2.1
(h) AN14670	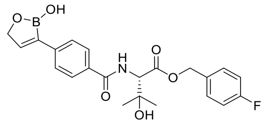	70.89 ± 51.72	372.3 ± 70.0	5.3	279.4 ± 12.8	3.9

(i) AN14839	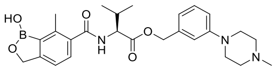	0.23 ± 0.16	2.12 ± 1.22	9	4.01 ± 2.67	18
(j) AN14772	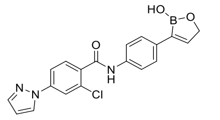	21.06 ± 6.51	32.32 ± 6.7	1.5	20.43 ± 2.51	1.0
(k) AN14955	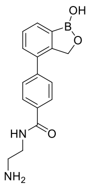	87.08 ± 21.87	101.2 ± 23.6	1.2	66.76 ± 40.30	0.8
(l) AN6311	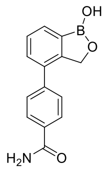	84.88 ± 21.31	141.9 ± 105.7	1.7	69.13 ± 24.74	0.8
(m) AN11912	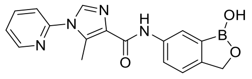	93.17 ± 37.93	83.62 ± 6.86	0.9	95.93 ± 26.08	1.0
(n) AN11909	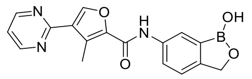	39.88 ± 11.21	53.05 ± 12.14	1.3	60.13 ± 19.81	1.5
AN11736	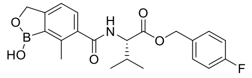	0.16 ± 0.10	20.85 ± 6.12	133	41.27 ± 16.62	264
Diminazene		248.1 ± 62.2	256.2 ± 62.7	1.0	223.1 ± 42.8	0.9

RF, resistance factor (ratio of the EC_{50} measured for the AN11736 resistant clones to the EC_{50} measured for the parental WT line, TcoWT). Data on the unrelated drug diminazene are also reported as control. The resistant clone derived from line B ($TcoOX^R\_B^{\#}$) used in this series of

experiments was different from the one ($TcoOX^R$_B) we refer to in the rest of the manuscript. Data represent means ± SD of $n \geq 3$ independent biological replicates.
