# Supplementary material for: Veterinary trypanocidal benzoxaboroles are peptidase-activated prodrugs
Source: PLoS Pathog. 2020 Nov 3;16(11):e1008932. doi: 10.1371/journal.ppat.1008932 (PMC7710103; doi:10.1371/journal.ppat.1008932)
Supplement: S3 Table — (PDF) [file ppat.1008932.s009.pdf]

Compound	TbWT	$TbOX^R$_A	
	EC_{50} nM	EC_{50} nM ± SD	RF vs WT
AN11736	0.20 ± 0.04	51.13 ± 2.71	259
Acoziborole	382.87 ± 25.30	218.17 ± 24.45	0.6
Diminazene	56.10 ± 25.82	49.28 ± 8.77	0.9
Pentamidine	0.41 ± 0.18	0.53 ± 0.04	1.3
Isometamidium	59.60 ± 39.07	38.96 ± 14.16	0.7
(c) AN14353	0.07 ± 0.10	52.17 ± 4.41	757
(h) AN14670	14.65 ± 6.84	88.68 ± 18.84	6.1
(j) AN14772	6.56 ± 1.76	8.61 ± 2.30	1.3

RF, resistance factor (ratio of the EC_{50} measured for the AN11736 resistant clones to the EC_{50} measured for the parental WT line, TbWT). Data represent means ± SD of n = 3.
