# Supplementary material for: Veterinary trypanocidal benzoxaboroles are peptidase-activated prodrugs
Source: PLoS Pathog. 2020 Nov 3;16(11):e1008932. doi: 10.1371/journal.ppat.1008932 (PMC7710103; doi:10.1371/journal.ppat.1008932)
Supplement: S4 Table — (PDF) [file ppat.1008932.s010.pdf]

**Primers for integration into pRM481**	
Primer name	Sequence 5’ → 3’
Fw_TbCBP1B	GGGGTCTAGAATGATGCTGTGCCACACG
Rev_TbCBP1B	GGGGGGATCCTTACGAGAGTGGTTCGTT
Fw_TvCBP1	GCACTCTAGAATGCGTTTGATATCAACTTGCAC
Rev_TvCBP1	CATGGGATCCAGGAAGCGGCTTGTTTTTCATG
Fw_TcoCBP1A	CGTCTCTAGAATGAAAGTTATATCGTACCCCGTC
Fw_TcoCBP1H	CCTATCTAGAATGCGCACGCATATAACGTTAC
Rev_TcoCBP1A/H	GATCGGATCCCTGAAGTGGTTTATTCTTCATGAATG
**Primers for generation of *T. congolense* tubulin locus integration plasmid**	
Fw_5'_tubulin_IR T. congo	acggccagtgaattcttaattaaggcgcgccACGGCAGTTGCCGACGAA
Rev_5'_tubulin_IR T. congo	gcttggccatctcgagGGTGAATGAATAGGAGTGCTTTGTTG
Fw_3'_tubulin T. congo	gcgttaccgctaaggatccAGAACAGGAAAAGAAGTG
Rev_3'_tubulin T. congo	acgccaagcttttaattaaggcgcgccactagtGATGGAATTGGATTAGTCTTC
Fw_ actin_IR T. congo	gtgggagggctaaccatggTTACAACGTGTTGTATGG
Rev_actin_IR T. congo	cttgctcaccatatcgatTGTAGCGAAAGTGGTATTTC
Fw_BSD	ctattcattcaccctcgagATGGCCAAGCCTTTGTCTC
Rev_BSD	acacgttgtaaccatggTTAGCCCTCCCACACATAAC
Fw_GFP	cactttcgctacaatcgatatgGTGAGCAAGGGCGAGGAG
Rev_GFP	tttcctgttctggatccTTAGCGGTAACGCTTGTACAG

NOTE: Capitalised letters, sequence identical to the genetic element described by the primer name; small letters, sequence belonging to the plasmid backbone or the adjacent genetic element	
**Primers to insert into and modify *T. congolense* tubulin locus integration plasmid**	
Primer name	Sequence 5’ → 3’
GFP ClaI to SalI_1	TTTCGCTACAgtcgacATGGTGAGCA
GFP ClaI to SalI_2	GTGGTATTTCTTTTGGTAAATCGAATC
pTco_GFP_HpaI_1	gttaacAGAACAGGAAAAGAAGTGTG
pTco_GFP_HpaI_2	GGATCCTTAGCGGTAACG
pRM481_TcoCBP1A_1	gttaacAAGCTAATTCGAGCTCGGTAC
pRM481_TcoCBP1A_2	CCCGGGTCATCACGCATA
Fw2_TcoCBP1A	CGTCGTCGACATGAAAGTTATATCGTACCCCGTC
Fw2_TcoCBP1H	CCTAGTCGACATGCGCACGCATATAACGTTAC
Fw_TbCBP1B	CGTCGTCGACATGATGCTGTGCCACACGTC
Rev_TbCBP1B	GATAGGATCCCGAGAGTGGTTCGTTCCTC
