# Supplementary material for: Veterinary trypanocidal benzoxaboroles are peptidase-activated prodrugs
Source: PLoS Pathog. 2020 Nov 3;16(11):e1008932. doi: 10.1371/journal.ppat.1008932 (PMC7710103; doi:10.1371/journal.ppat.1008932)
Supplement: Attachment — Submitted filename: Plos response to referees2.docx [file ppat.1008932.s011.docx]

10th August 2020

Dear Plos Pathogens,

**Veterinary trypanocidal benzoxaboroles are peptidase-activated prodrugs: Response to referees**

We have now made the changes requested and outline those here, as well as attaching a marked up as well as clean version of the revised manuscript. Changes include:

1. All figures have been uploaded at the required reproduction level
2. In response to Reviewer #1:
1. line 116: "had increased"

We believe “decreased” was correct..i.e. sensitivity of parasites to drug had decreased (them being less sensitive) albeit that the IC50 had increased. We have thus kept “decreased” on line 116 (which is not line 129

2. line 128: the trypanosomes were resistant, not the compounds

We have changed this sentence to say (new line = 141):

“Further cross-resistance analysis in *T. congolense* using a diverse array of benzoxaboroles revealed that AN11736-resistant trypanosomes were cross-resistant to compounds with a peptide-bond linker containing a valinate-amide motif, whereas these parasites showed no cross-resistance to benzoxaboroles without the linker. “

3. line 428: what was the DMSO concentration?

We have added with the DMSO vehicle control (below 1% v/v) (now line 523)

4. figure 1, right panel: "% Survival" is not clear. Was this really a survival assay or should it read "% Growth"?

In fact we believe % survival to be okay in this case since the compound is trypanocidal. Although the assay measures metabolic conversion of resazurin to resorufin reagent and this can also be hindered by trypanostatic compounds we believe in this case it is a true surrogate for cell death, and add this to the legend to figure 1.

3. Reviewer 2 requested some additional information in the Discussion about roles for CBP1 serine carboxypepidase in T. brucei and whether it may have an essential role in procyclic forms. We do not know the answer to either question but have amended the text to address the issues:

“*It is probable that the T. brucei and T. congolense CBP serine carboxypeptidases play similar roles to that in T. cruzi where its lysosomal localisation and multiple hydrolytic capabilities [32,33] likely play a generic role in macromolecule turnover. We have not ascertained whether the genes are essential in procyclic form parasites which are resident in the tsetse fly. It will be important to understand this in the future since a fitness cost in this lifecycle stage would hinder the transmission of parasites that develop resistance via this route in the mammalian bloodstream*.”

4. Reviewer 2 also asked to clarify the final sentence of the Discussion given the possibility that if linkers were added to other potential prodrugs to enable activation inside the parasites they would suffer a similar risk of resistance in peptidase resistance parasites. We have therefore changed that last sentence to say:

“*As well as elucidating the resistance mechanism to a class of potent benzoxaboroles, the discovery of a particular moiety that is specifically cleaved by trypanosomal carboxypeptidases offers the potential to exploit that linker to create novel prodrugs with targeted activity against trypanosomatids, i.e. drugs that may not have the ability to traverse membranes could be linked, via the CBP cleavable bridge to hydrophobic moieties enabling diffusion into cells where they would be cleaved to release the specific inhibitor. A risk of resistance to such compounds emerging through mutation to the CBP genes could limit such a use*.”

**Change in author order:**

In addition to these requested changes, we have also changed the position of the two joint first authors such that Giordani is now ahead of Paape.

Yours Sincerely
